# Thoracoabdominal normothermic regional perfusion: Is it ethical?

**DOI:** 10.1007/s40592-025-00229-2

**Published:** 2025-01-29

**Authors:** Caner Turan

**Affiliations:** Faculty of Arts and Sciences, American University of Beirut, Riad El Solh, Beirut, 1107 2020 Lebanon

**Keywords:** Thoracoabdominal normothermic regional perfusion (TA-NRP), Controlled donation after circulatory death (DCD), The dead donor rule, The uniform determination of death act (UDDA), Respect for persons, The doctrine of double effect

## Abstract

Thoracoabdominal normothermic regional perfusion (TA-NRP), a new method of controlled donation after circulatory death, seems to provide more and better organs for patients on organ transplant waiting lists compared to standard controlled donation after circulatory death. Despite its benefits, the ethical permissibility of TA-NRP is currently a highly debated issue. The recent statement published by the American College of Physicians (ACP) highlights the reasons for these debates. Critics’ main concern is that TA-NRP violates the Dead Donor Rule. This paper presents an ethical analysis of the objections raised by the ACP against TA-NRP and argues that TA-NRP is not only morally permissible but also morally required where it is financially and technically feasible. To support this conclusion, the concepts of ‘resuscitation,’ ‘intention,’ ‘irreversibility,’ ‘permanence,’ ‘impossibility,’ and ‘respect’ in the context of TA-NRP are explored. Additionally, the ethical permissibility of this procedure is evaluated through the lenses of Utilitarianism, Kantianism, the core principles of bioethics, and the Doctrine of Double Effect. This ethical analysis demonstrates why the ACP’s objection lacks a solid moral foundation and conflates moral and legal considerations. This paper also argues that extra measures are needed to ensure the moral permissibility of TA-NRP, emphasizing the importance of informed consent, additional brain blood flow and activity monitoring, and a contingency plan to abort the organ procurement process if a sign of morally relevant brain activity is detected.

The acceptability of organ donation after circulatory death (DCD) varies culturally and internationally. While many European countries actively practice DCD, some do not permit it at all, and even in countries where it is allowed, there are stricter regulations for its use in children. Additionally, some European countries are planning to start DCD programs (Lomero et al. 2019). Overall, the trend indicates that DCD is gaining more acceptance in Europe. There is also a global increase in the number of countries adopting policies to promote DCD. For example, the use of DCD has been rising in North America, Latin America, and Australia (Bendorf et al. 2013; Langer 2023).

There is greater ethical debate and variability in the acceptability of thoracoabdominal normothermic regional perfusion (TA-NRP), a new method of donation after circulatory death (Brierley et al. 2024). Even though TA-NRP is gaining popularity due to its benefits over standard DCD, there is uncertainty regarding its ethical permissibility, with many national ethics bodies currently debating the issue. In some countries, such as Australia, TA-NRP is not permitted even though DCD is allowed. In Europe, there is also less acceptance and use of TA-NRP compared to DCD (ibid.). In the USA, the use of TA-NRP has been increasing since its inception in 2020 (Bekki et al. 2023). However, there is ongoing debate about the ethical implications of this practice. The American College of Physicians (ACP) expressed these ethical concerns in a recent statement and urged that this new method be stopped. This statement serves as a very suitable starting point, as it sheds light on ethical discussions concerning TA-NRP around the world.

This paper offers an ethical analysis of the ACP’s objections to TA-NRP, arguing that the use of TA-NRP is ethically permissible with certain provisions and adheres to all moral principles in bioethics. My conclusion goes further: TA-NRP is not only morally permissible but also required where it is financially and technically feasible. To establish this conclusion on a solid foundation, I will first discuss the practical differences between standard DCD and TA-NRP (Sect. 1). Following this, I will explain why the ACP finds TA-NRP ethically problematic (Sect. 2).

A significant portion of this paper is devoted to Sect. 3, where I examine the ACP’s objection that TA-NRP breaches the Dead Donor Rule by resuscitating the patient and causing brain death. Here, I will explore the concepts of ‘resuscitation,’ ‘intention,’ ‘irreversibility,’ ‘permanence,’ ‘impossibility,’ and ‘respect’ in the context of TA-NRP, demonstrating why the ACP’s objection lacks a solid moral foundation and conflates moral and legal considerations. The claims I defend here are: (1) the ACP fails to provide any compelling reason to consider TA-NRP morally inferior to standard DCD; (2) TA-NRP does not lead to resuscitation or cause brain death, nor is there any intention or attempt to do so during the procedure; (3) clinically insignificant circulatory activity or function after death declaration, or the theoretical possibility that TA-NRP accelerates brain death, does not make TA-NRP morally impermissible; (4) even when brain death is accelerated during TA-NRP, one might view this as an act of non-maleficence; (5) neither the ACP nor other critics of TA-NRP can appeal to the Doctrine of Double Effect to show a moral difference between standard DCD and TA-NRP; and (6) if TA-NRP is ethically permissible or required, legal regulations and policies should adapt accordingly.

In Sect. 4, after outlining the measures needed for TA-NRP to fully comply with bioethical principles, I will address the ACP’s remaining objections. Here, considering the theoretical possibility of morally relevant brain activity (e.g., awareness or pain perception), I will argue that additional brain blood flow and activity monitoring and a contingency plan to abort the organ procurement—if a sign of such activity is detected—may be necessary for both TA-NRP and standard DCD. Moreover, I will stress the importance of providing patients and/or surrogates with detailed yet simplified information about the procedure and the necessity of obtaining informed consent. I will argue that applying the ‘right not to know’ to organ donation may lead to a greater violation of the principle of respect for autonomy than not applying it. Finally, I will claim that TA-NRP does not create an extra justice problem beyond existing inequities, and that ex situ perfusion techniques increase the costs of organ procurement and are less effective at protecting organs than TA-NRP.

## The procedures: TA-NRP vs. standard DCD

There are two main types of organ donation: (1) donation after brain death (DBD)—irreversible loss of all brain functions, including the brain stem—and (2) donation after circulatory death (DCD)—irreversible loss of all circulatory and respiratory functions. Although DBD is more common, the number of DCD cases has been increasing due to technological advances, changes in policies, and a growing social acceptance of DCD.

The predominance of DBD is because there is no loss of circulation, allowing the organs to receive oxygen and be more easily protected compared to DCD, where the lack of oxygen leads to organ damage (ischemia), thus reducing the number of organs suitable for donation. In DCD, this issue is being addressed through the use of machine perfusion, a method that involves perfusing organs with a preservation solution to minimize damage from ischemia and maintain organ viability. This improvement leads to a higher number of functioning organs available for donation, which is excellent news for patients on organ transplant waiting lists.

However, machine perfusion does not always perfectly preserve organs. Organs such as the heart and intestines, which are more susceptible to ischemic injury, exhibit significantly lower success rates in DCD compared to other organs. The technique of thoracoabdominal normothermic regional perfusion with controlled^1^ donation after circulatory death (TA-NRP) is gaining popularity because it substantially increases these success rates. This new method involves restarting the patient’s circulation and supplying the thoracic and abdominal organs with the patient’s own blood at body temperature, drastically improving organ quality compared to standard DCD. For example, TA-NRP can lead to better outcomes in liver, kidney, and pancreas transplantation (Bekki et al. 2023).

In both standard DCD and TA-NRP, life-sustaining therapies are withdrawn at the request of the patient and/or their surrogate(s). A physician then declares the patient dead based on circulatory criteria (irreversible cessation of all circulatory and respiratory functions), after a ‘hands-off’ period of varying length (typically, five minutes) to make sure there is no auto-resuscitation.

In standard DCD, physicians make incisions to access organs within chest cavity and abdomen. The abdominal aorta is then clamped to direct the flow of a preservative solution to the abdominal organs. Typically cold, this solution is used to reduce the metabolic rate of the organs, thereby decreasing their oxygen demand and preserving their function until transplantation (James et al. 2022b). By stopping the flow of blood, and substituting it with a preservative solution, the organs are protected from the detrimental effects of oxygen deprivation during the period between procurement and transplantation.^2^

In TA-NRP, physicians make the same incisions as in standard DCD, but instead of cooling the body, they first prevent blood from flowing to the brain by blocking the arteries that supply blood to the head and neck. Next, the patient’s blood is circulated through a machine (CPB or ECMO), which pumps and oxygenates the blood, delivering it to the thoracic and abdominal organs at body temperature (Wall et al. 2022). Restoring circulation significantly reduces the time that organs are deprived of oxygen, thereby substantially improving the quality of the organs (Lazaridis 2022). This results in more and better functioning organs being available for transplant.

## The objections by the ACP

Since TA-NRP improves the quality and quantity of transplanted organs, it provides better patient outcomes compared to the standard DCD. Despite its benefits, the American College of Physicians (ACP) wants TA-NRP to stop due to four ethical objections:

First, the ACP (2021) asserts that TA-NRP breaches the Dead Donor Rule and the Uniform Determination of Death Act (UDDA). The Dead Donor Rule prohibits organ procurement before the patient’s death and the procedure causing the patient’s death (Peled et al. 2022), while the UDDA requires *irreversible* cessation of all circulatory and respiratory functions *or* all brain functions to declare death (DeCamp et al. 2022a). From the ACP’s standpoint, a patient may initially be declared dead based on accepted criteria; however, restoring circulation challenges the validity of this declaration. The circulatory criterion stipulates that the cessation of circulatory functions must be ‘irreversible.’ When circulation is restarted, it implies that the cessation was reversible, thus negating the original declaration. Therefore, restarting circulation essentially amounts to ‘resuscitating’ the patient.

Furthermore, the ACP argues that when physicians intentionally block the arteries to prevent brain reperfusion, they are artificially inducing brain death. This indicates that, in the view of the ACP, brain death in TA-NRP does not occur naturally. The ACP believes that physicians manipulate the circumstances to conform to legal organ donation criteria,^3^ which, in their view, signifies a lack of respect for human dignity and reduces patients to *mere* means for organ donation.

Second, according to the ACP, a lack of transparency and informed consent regarding the TA-NRP protocol can harm trust in healthcare and clinical research. Moreover, even with informed consent, ethical legitimacy is not granted since medical ethics standards cannot be overridden. Autonomy has its limits, and informed consent alone does not necessarily confer ethical permissibility to actions.

Third, the ACP contends that TA-NRP contravenes the principle of equitable distribution of benefits and burdens, disproportionately affecting marginalized populations, particularly victims of drug overdoses. Such individuals often sustain severe brain damage without being declared brain dead, making DCD a prevalent method for them. However, expanding DCD could disproportionately increase their burden of organ donation. Given that victims of drug overdoses typically come from lower socioeconomic backgrounds and face numerous challenges, enhancing DCD in these cases could disproportionately affect this already marginalized group struggling with substance abuse.

Fourth, ex situ alternatives to in situ TA-NRP, which use machines external to the body for organ reperfusion, are deemed morally preferable by the ACP as they distinguish morally between “perfusing an organ versus perfusing an individual” (ACP 2021, 4). The rationale appears to be that using external machines eliminates the need to occlude cerebral circulation and restart circulation, thereby sidestepping the ethical controversies inherent in TA-NRP.

## The first objection: beneficence vs. respect

The ACP’s foremost concern regarding the breach of the Dead Donor Rule and the UDDA centers on two main issues related to the concepts of irreversibility and respect. First, a death declaration based on the circulatory criterion presupposes an irreversible cessation of circulatory and respiratory functions, implying that the circulation has stopped forever. However, this irreversibility is called into doubt when TA-NRP restores circulation for organ preservation. Therefore, restarting circulation seems to violate UDDA’s requirement of declaring death according to circulatory or brain death criteria, both of which are based on the irreversible cessation requirement. Moreover, this practice appears to infringe upon the Dead Donor Rule’s stipulation that organ transplantation occurs post-mortem, because the initial death declaration is rendered invalid, and accordingly, restoring circulation amounts to resuscitation. This raises concerns about respecting the patient’s dignity.

Second, when death is declared according to the brain death criterion (irreversible cessation of all brain functions, including the brainstem), it implies that all brain functions have ceased completely and forever. During TA-NRP, however, physicians actively intervene to obstruct reperfusion to the brain, indicating a concern that some brain activity may resume if blood flow is reestablished. This situation leads to the question: How can we claim irreversible cessation of all brain functions if we must actively prevent their potential resumption? Additionally, the ACP believes that occluding cerebral circulation goes against the natural progression of death. This practice seems to violate the Dead Donor Rule’s mandate that the process of organ procurement should not cause the donor’s death. Knowing that circulation will be restarted, and unable to depend on the circulatory criterion of death, physicians aim to ensure that brain death occurs, allowing organ procurement based on the brain death criterion. The ACP (2021, 4) views this as treating the patient solely as a means to an end—organ donation—and as a violation of their dignity, which cannot be justified by the principle of beneficence or the utility of organ donation.^4^

If we are to consider stopping the use of TA-NRP, we must establish a significant moral distinction between TA-NRP and standard DCD. The ACP does not raise ethical objections to standard DCD, yet finds TA-NRP morally problematic. Thus, examining this distinction is crucial. Should TA-NRP prove morally inferior to standard DCD, stopping its use might be justified. If this new method inherently disrespects human dignity, its additional utilitarian benefits may not alone render it ethically acceptable. But is there truly a morally relevant difference between these two procedures?

### Does restoring circulation amount to resuscitation?

When examining the criticisms of TA-NRP by some opponents, it appears they believe physicians intend to resuscitate the patient using this method. For instance, when discussing standard DCD, the ACP states that it conforms to the ‘irreversible cessation’ requirement “because there is *no intention* to resuscitate and auto-resuscitation is not possible [which is confirmed by the hands-off period]” (ACP 2021, 1; emphasis added). Since they believe (i) auto-resuscitation is not possible in either standard DCD or TA-NRP, and (ii) TA-NRP does not meet the ‘irreversible cessation’ requirement, this seems to imply that they also believe there *is* an intention to resuscitate associated with TA-NRP. Some other detractors oppose TA-NRP because “the intention from the outset is to restart circulation” (DeCamp et al. 2022a, 288). While it is true that restarting circulation is an integral part of TA-NRP, the moral implications become significant if there is also an intention to resuscitate the patient. This would be highly problematic morally, as the Dead Donor Rule prohibits organ retrieval from a donor who is not already deceased or who is resuscitated.

This explains why, when some supporters of TA-NRP assert that both standard DCD and TA-NRP involve the same premortem interventions and adhere to the UDDA’s circulatory criterion (James et al. 2022a), their argument is met with skepticism. According to these supporters, a hands-off period confirms the absence of spontaneous function and indicates natural death, suggesting no distinction between the two methods (Adams et al. 2022). Despite this, detractors remain unconvinced. Detractors’ concerns lie not in the premortem interventions or initial adherence to the circulatory criterion, but in their claim that restoring circulation after a circulatory death declaration contradicts the declaration itself and essentially equates to resuscitation. Consequently, they find the argument that the patient was already dead to be inadequate (DeCamp et al. 2022a, 289).

Some supporters of TA-NRP respond to this claim by asserting that physicians intend to perfuse organs for transplantation rather than resuscitate the donor in TA-NRP, as resuscitation attempts would be futile. This makes the act of restoring circulation distinct from resuscitation (Parent et al. 2022, 1308). To evaluate the plausibility of this claim, it is necessary to understand what resuscitation means. According to the Oxford English Dictionary, resuscitation is “the action of restoring a person to life after death” or “emergency treatment designed to restore circulatory and respiratory function after cardiac arrest, major trauma, etc.”^5^ It is true that circulation is restored in TA-NRP, but (a) it is not an emergency treatment (it is not a treatment at all), and (b) it does not result in bringing a person ‘back to life’ after death. Given today’s medical technology, it is simply unachievable to revive a patient from unconsciousness or death after observing a hands-off period of five minutes and declaring the patient dead.^6^ Additionally, once the decision to donate the patient’s organs has been made, the focus shifts to organ donation instead of patient revival. In medical practice, resuscitation refers to the emergency interventions aimed at restoring and supporting vital functions such as heartbeat and respiration in life-threatening circumstances, often requiring continued medical intervention beyond the initial resuscitative efforts to keep the patient alive and functioning. Given that resuscitation typically involves significant medical intervention to restore and support vital functions, and considering that such comprehensive restoration is unachievable in TA-NRP, how, then, can physicians intend to resuscitate the patient in TA-NRP when they are well aware that this will not be the case?

If an aim is known to be unachievable, it seems impossible to genuinely intend that aim in a rational manner. Consider an astronomer who recognizes the unattainability of traveling to a galaxy millions of light-years away within a lifetime with existing technology. While this astronomer can certainly dream of undertaking such space travel, they cannot truly intend it. Similarly, a person without any experience or training in tennis cannot genuinely intend to win a Grand Slam within a year, provided they have an accurate judgment and perception. They might wish for magical powers to achieve this, but they cannot form a true intention. No competent human being can rationally intend to fly like birds, given our inherent incapacity for flight. In the same vein, a competent physician, aware that resuscitation is unachievable during TA-NRP, cannot rationally intend to resuscitate anyone.

D. M. Armstrong acknowledges this point in his “Meaning and Communication,” where he differentiates between objectives and intentions. He argues that having an intention is a narrower concept than having an objective. More precisely, intentions involve a person’s firm commitment to perform a specific action and include a belief in one’s ability to carry out the action, assuming no unforeseen circumstances prevent it. He notes that “expression of intentions entails that the speaker believes that the thing aimed at is *within his power*, an entailment that is absent in the expression of mere objectives” (Armstrong 1971, 432n.2; emphasis added). That is, unlike objectives, which do not inherently include a belief or assurance in one’s capability to achieve A, intentions must include a belief in one’s ability to achieve A.^7^

To apply this reasoning to physicians’ mental states during TA-NRP, it seems that physicians cannot truly intend to resuscitate the patient when they restore circulation. Some physicians may hold a broader objective to make resuscitation feasible in situations where it is impractical with today's medical technology, even when they are uncertain of their success. They can engage in or support research aimed at advancing resuscitation technologies and techniques, participate in clinical trials that test new methods or technologies, engage with policymakers to advocate for research funding, and contribute to or partner with tech companies developing medical devices that enhance resuscitation outcomes. However, possessing this broader objective in no way means these physicians have the objective or intention to resuscitate the patient in a specific instance of TA-NRP. Considering that restarting circulation to allow the patient’s own blood to flow at body temperature significantly protects organs, their objective—and their intention—is more likely to be to procure better and more organs for transplantation.^8^

The ACP would likely not be persuaded by the above line of reasoning because, later in their statement, they seem ready to concede to the supporters of TA-NRP that the primary intention of physicians during TA-NRP may indeed be to preserve more and better organs and save more lives. However, according to them, this does not alter the fact that resuscitation occurs in TA-NRP: “Even if the stated intent of NRP-cDCD [TA-NRP] is to preserve the organs (not to resuscitate the patient), this obscures and cannot be separated from what actually happens: NRP does resuscitate the patient” (ACP 2021, 3). This seems to contradict their earlier implication that physicians *do* intend to resuscitate patients in TA-NRP, but I will leave this matter aside. Regardless of the ACP’s position on physicians’ intentions, the main point of the ACP appears to be that intentions are *irrelevant* when determining the validity of a death declaration. Rather, it is the biological or physical facts that dictate the validity. The point is simple: physicians declare someone dead based on the cessation of circulation, then they restart the circulation, thereby making that declaration invalid. Regardless of what physicians intend, this is indeed what actually happens.

The ACP’s position on the irrelevance of intentions in determining the validity of a death declaration and their adoption of the biological or physical conception of irreversibility are also shared by other critics of TA-NRP. They claim that while intentions may be relevant to the moral assessment of physicians’ actions, they do not factor into the *legal* criteria for declaring death (Glazier and Capron 2022). In TA-NRP, “[t]he lack of intent to resuscitate the patient […] does not change the biological reality” that circulation first stops and then resumes (Peled et al. 2022, 1647). Therefore, from a legal standpoint, factors such as intentions, the absence of a meaningful existence, the medical futility of potential resuscitation attempts, and even the patient’s consent “are all irrelevant to whether the patient is deceased under US law, which turns on the person’s physical condition not on anyone’s intention” (Glazier and Capron 2022). For example, if auto-resuscitation occurs after the hands-off period and just before organ procurement in standard DCD, physicians must stop the procedure because the prior declaration of death is invalidated. When that happens, it does not necessarily indicate that the patient was dead and then raised from the dead; it merely demonstrates that the prior declaration of death was incorrect. The same is true for TA-NRP (DeCamp et al. 2022b).^9^

To sum up, the ACP initially identifies two conditions for the irreversibility of cessation in circulatory or respiratory functions: (1) auto-resuscitation is not possible, and (2) there is no intention to resuscitate. As previously argued, physicians cannot intend to resuscitate the patient during TA-NRP because (a) they know that reviving the patient after the hands-off period with today’s technology is unachievable, and (b) one cannot intend what one knows to be unachievable. Even if one were to refute (b), it would still be far-fetched to claim that physicians actually intend to resuscitate the donor during TA-NRP, considering their inaction during the hands-off period and their awareness that the process aims to procure organs from a dead donor rather than to revive the donor. (So, no attempts are made to restart the heart for the purposes of saving or prolonging life.) However, we have observed that the ACP contends the moral issue with TA-NRP does not lie in the physicians’ intentions but rather in the physical condition of the patient: “the patient is, in fact, successfully resuscitated” (ACP 2021, 2). Thus, the revised conditions for irreversibility are: (1) auto-resuscitation is not possible, and (2) the patient is not resuscitated.

At first glance, the first condition appears to be satisfied in both procedures because ACP (2021, 1) states that the impossibility of auto-resuscitation is confirmed by the absence of auto-resuscitation during the typical five-minute hands-off period, observed in both procedures. Therefore, this condition does not seem to provide a moral or other basis for differentiating between standard DCD and TA-NRP.

Nevertheless, one may rightly argue that ACP’s stance on the ‘impossibility’ of auto-resuscitation is not entirely accurate. Some opponents of the TA-NRP have mentioned the possibility of auto-resuscitation following a hands-off period of five minutes, as previously noted. They have argued that if auto-resuscitation occurs, the procedure should be aborted (DeCamp et al. 2022b). The unassisted return of spontaneous circulation after cardiac arrest, known as the ‘Lazarus phenomenon,’ is not impossible after the five-minute hands-off period, although it is highly unusual. Indeed, there are recorded cases of functional recovery following auto-resuscitation that occurred after this period (Sahni 2016).

If auto-resuscitation is not ‘impossible’ in both procedures, even after the hands-off period of five minutes, then standard DCD also does not meet the conditions of irreversibility adopted by the ACP and other critics, and thus it should be stopped as well. After all, according to the critics, intentions and consent are irrelevant when determining the validity of a death declaration, and we should consider the issue from a purely physical or biological standpoint. But this is clearly not the direction the ACP wishes to take. What goes wrong in the opponents’ reasoning?

I believe that focusing on the physical or biological conception of irreversibility leads to an expansive understanding of resuscitation that is legally—but not morally—relevant. According to this understanding, a patient is considered resuscitated (a) if the patient is brought back to life, implying some level of sentience or consciousness, and/or (b) if the declaration of death is invalidated. The latter, based on the irreversible cessation requirement of the UDDA, pertains to the legal aspect of resuscitation, but its violation is not necessarily morally wrong.

Some violations of the irreversibility condition, including those in standard DCD and TA-NRP, may be morally permissible from both utilitarian and Kantian perspectives, and according to the core principles of bioethics.^10^ For instance, if some cardiac electrical activity or agonal contractions occur during or after the hands-off period, and if these circulatory functions are not sufficient to support vital body functions and to restore some level of consciousness or sentience, it seems *morally* permissible to continue with the procedure (standard DCD or TA-NRP), provided that informed consent has been obtained, unless a compelling reason suggests otherwise.^11^ First, a mere physical/biological change does not necessarily constitute a moral change. If the return of circulatory function resulted in some level of pain perception, for example, then continuing with the procedure would be morally wrong due to the violation of the principle of non-maleficence. However, if this biological change does not result in any level of sentience or consciousness, where the principles of beneficence and non-maleficence do not apply, continuing with the procedure seems morally permissible from a utilitarian perspective, although it may be considered a legal violation.

Moreover, a mere legal violation does not necessarily imply that one person is using another merely as a means in the Kantian sense. Violating informed consent procedures might amount to disrespecting one’s autonomy and using them as a mere means; however, it is not easily argued that procuring organs after a clinically insignificant circulatory activity following hands-off period or restoring circulation in TA-NRP constitutes a violation of the principle of respect for autonomy, assuming that informed consent has been obtained. According to Kant, human dignity is rooted in our capacity to determine our life’s trajectory. “*Autonomy* is therefore the ground of the dignity of human nature,” which holds an “unconditional, incomparable worth” (*Groundwork of the Metaphysics of Morals*, 4:436).^12^ For this reason, treating someone as a mere means can be characterized by intentionally using them for one’s own purposes while disregarding their actual (and informed) consent (Parfit 2011: 221–28; Kleingeld 2020).^13^ Hence, while the requirement to obtain informed consent is a legal rule whose violation *prima facie* amounts to using someone as a mere means, the breach of a legal rule that prohibits procuring organs from a deceased body (in a morally relevant sense, not legally) *prima facie* does not amount to using someone as a mere means.^14^ One may even argue that compliance with this law in some cases is morally objectionable, considering the benefits of organ donation.

Opponents of TA-NRP must provide a moral reason (as opposed to a legal one) why the continuation of the organ procurement process should be considered impermissible in the event of a clinically insignificant circulatory activity during the procedure. Firstly, as argued, if the resumption of circulatory function after death declaration does not result in any level of consciousness or sentience, there is no utilitarian basis to claim that continuing with the procedure is morally impermissible. Accordingly, bioethical principles of beneficence and non-maleficence are irrelevant. Secondly, the principle of the sanctity of life cannot be invoked, as we cannot consider a ‘life’ at this point, which has permanently ended. Furthermore, if the donor or their family has given informed consent to the organ donation procedure, particularly regarding the continuation of the procedure under these conditions, it is not plausible to claim a violation of the principle of autonomy. In the absence of any compelling moral reason to stop the procedure once such activity is observed, it falls to the opponents to present a moral consideration that we may have overlooked, without confusing moral with legal considerations.

The discrepancy between moral and legal considerations becomes conspicuous when discussing our treatment of animals. There has been considerable debate about the moral status of animals and how we treat them. For example, even though we can find strong utilitarian (and even Kantian)^15^ grounds against torturing animals in factory farms, many countries lack meaningful legislative protections for farmed animals (Kim 2022). To give a more specific example, many people enjoy boiling lobsters alive; however, recent research suggests that decapod crustaceans such as shrimp, lobsters, crayfish, and hermit crabs can experience feelings, including pain (Conte et al. 2021). What, then, is the moral difference between boiling a lobster and boiling a dog, given that both can feel pain? Many are deeply troubled by this practice and have empirical and moral reasons to advocate for making it illegal. Although boiling lobsters alive is legal almost globally,^16^ it seems morally objectionable if lobsters are capable of feeling pain. In contrast, finding a valid moral reason to halt the organ procurement process due to clinically insignificant circulatory activity during the procedure proves much more difficult, for the reasons mentioned above. Nonetheless, if legal regulations obstruct such procedures, the critique should be directed at the legal systems, not at our moral reasoning.

One might argue that violating legal rules itself is morally problematic, regardless of the specific rules being violated. This claim could be supported by two reasons. First, adhering to legal rules might inherently constitute a moral obligation. Second, toleration of minor violations of legal rules could encourage more significant breaches of law, the harms of which would far outweigh the benefits derived from TA-NRP. Accepting the first reason could justify *any* action as long as it is legal. From this perspective, practices such as slavery, torture, female genital mutilation, disenfranchisement of women, stoning, or flogging would be deemed morally permissible where they are legal. The second argument, on the other hand, loses its force considering our ability to reform laws to endorse ethically acceptable actions. This has been the historical practice. Sometimes, immoral actions are made illegal, as many countries, including the US, have done with slavery. Conversely, some countries have legalized actions they consider *prima facie* morally permissible, such as abortion, the consumption of alcohol or marijuana, and same-sex marriages.

A similar approach could be applied to death declaration policies. One interesting proposal is to replace the term ‘irreversible’ with ‘permanent’ (Parent et al. 2022, 1308). Under this revision, the loss of circulatory functions would be considered permanent after cardiac arrest when (1) there will be no auto-resuscitation,^17^ and (2) there is no intention or attempt to resuscitate the patient.^18^ Provided that restoring circulation during TA-NRP meets both conditions, it would be legally and morally permissible. The key is that circulation has ‘permanently’ ceased, not that it is absolutely impossible to restart it or for some clinically insignificant circulatory activity to return. In theory, there is always a minimal chance that some clinically insignificant bodily functions could unexpectedly return temporarily or be temporarily restored through resuscitation efforts. Therefore, it is sufficient for the cessation of circulation to be considered ‘permanent’ if physicians agree to cease resuscitation attempts, acknowledging the theoretical possibility that some temporary, clinically insignificant activity might unexpectedly return.^19^ Conversely, applying the ‘irreversibility’ condition could hinder physicians from declaring anyone dead due to the possibility of the resumption of a minimal amount of function temporarily, which does not align with standard medical practice. Since moral considerations are the foundation for establishing legal rules, not vice versa, and considering that the ACP grounds its argument in legal rather than ethical considerations by conflating the morally and legally relevant meanings of ‘resuscitation,’ their ethical conclusion does not hold a base.

What about the cases where patients survived following auto-resuscitation that occurred after a hands-off period of five minutes? Should we reconsider the duration of this period? In fact, many studies have indicated that a five-minute hands-off period is adequate for controlled organ donation. A 2021 study found that 14% of patients had transient cardiac activity after pulselessness, with only 1% detected at bedside, all occurring within 4 min and 20 s. The median duration of resumed cardiac activity was 3.9 s, lasting only one cardiac cycle in 49% of cases, indicating a lack of clinical significance (Dhanani et al. 2021).

Furthermore, a systematic review of studies reported that out of 1049 patients, 19 instances of auto-resuscitation were noted, constituting approximately 1.8% of the cases. In all these cases, the heart started beating again within five minutes of cessation, but none of these patients ultimately survived. According to this review, five minutes is an acceptable hands-off period to declare circulatory death in the context of controlled organ donation, and for uncontrolled organ donation, an observation time greater than five minutes may be needed (Zorko et al. 2023). The previously mentioned review discussing functional recovery cases following auto-resuscitation reports that, in most cases, auto-resuscitation has been observed within 10 min and recommends a hands-off period of 10–15 min after the withdrawal of CPR (Sahni 2016).^20^ So, this duration could possibly be suitable for uncontrolled organ donation.

My aim is not to prescribe the appropriate duration of the hands-off period in controlled and uncontrolled organ donation settings, nor am I in a position to do so. The proposed durations may change as further evidence emerges or additional research is conducted. Rather, my point is that, regardless of the established duration for the hands-off period in TA-NRP, physicians should abort the procedure if the circulatory activity during or after this period is clinically meaningful. If the circulatory activity during and after hands-off period is not clinically meaningful, then it seems morally permissible to continue with TA-NRP procedures, irrespective of the provisions of the UDDA. This should not be interpreted as a recommendation to perform illegal actions. However, if the UDDA prevents physicians from performing actions that are morally permissible and beneficial, it suggests that the UDDA requires revision. If this is indeed the case, we should not stop TA-NRP; instead, we should revise the UDDA.

## Does occluding cerebral circulation amount to causing brain death?

My argument so far is as follows:

*Premise 1*. During an organ donation procedure, if the resumption of circulatory activity or function after death declaration is clinically insignificant and informed consent has been obtained regarding the procedure and the withdrawal of life-saving treatments, then the procedure is morally permissible.

*Premise 2*. In TA-NRP, the resumption of circulatory activity or function after death declaration is clinically insignificant.

*Premise 3*. In TA-NRP, informed consent has been obtained regarding the procedure and the withdrawal of life-saving treatments.

*Conclusion*. Therefore, TA-NRP is morally permissible.

My focus has been primarily on the first premise. My goal has been to explain why, in the context of organ donation, if the resumption of circulatory activity or function after a death declaration is clinically insignificant, the procedure is morally permissible, provided that informed consent has been obtained.^21^ Although opponents of TA-NRP seem to acknowledge the truth of the second premise—and arguably the third premise as well—they introduce an additional condition to the first premise: ‘the procedure is morally permissible if the UDDA’s provisions for the declaration of death are fulfilled throughout the procedure.’ They thereby argue that the invalidation of the initial declaration of death in TA-NRP renders the procedure morally impermissible. I have countered that their view is rooted in legal rather than moral concerns and carries problematic implications, such as the idea that legal standards dictate moral standards. I have maintained that the moral weight of the first premise should guide legal standards, not the other way around.

However, this is only one aspect of the ACP’s critique of TA-NRP. Even if we accept the second premise, we must further inquire *why* the resumption of circulatory activity or function after death declaration is clinically insignificant in TA-NRP. According to the ACP, this occurs because physicians block the arteries to prevent brain circulation, which “renders the patient brain dead so that circulation can be restored and the patient still be considered dead” (ACP 2021, 2–3). The ACP believes that “[b]rain death has been caused in order to prevent brain reperfusion when circulation is restored” (ibid., 3). In their view, the aim of this is to justify reversing circulatory death, which was previously deemed irreversible.

I do not believe it would be accurate to say that occluding cerebral circulation in TA-NRP *causes* brain death because brain death is inevitable after circulatory death. A more appropriate way to express this concern, which is also echoed by the ACP, is to state that preventing brain reperfusion in TA-NRP goes against the *natural* course of death. In other words, TA-NRP, unlike standard DCD, *artificially hastens* the brain’s dying process. The ACP finds this morally troublesome because it views the artificial hastening of brain death as using the patient merely as a means to the end of restoring circulation and securing more and better organs. Therefore, the ACP proposes yet another condition for the moral permissibility of organ donation procedures: ‘the procedure is morally permissible if brain death is not artificially hastened during the procedure.’ Let’s accept this condition for the sake of the argument. If we are to defend the ethical permissibility of TA-NRP, the revised argument should be:

Premise 1*. During an organ donation procedure, if the resumption of circulatory activity or function after death declaration is clinically insignificant, informed consent has been obtained regarding the procedure and the withdrawal of life-saving treatments, and brain death is not artificially hastened during the procedure, then the procedure is morally permissible.

*Premise 2*. In TA-NRP, the resumption of circulatory activity or function after death declaration is clinically insignificant.

*Premise 3*. In TA-NRP, informed consent has been obtained regarding the procedure and the withdrawal of life-saving treatments.

*Premise 4*. In TA-NRP, brain death is not artificially hastened.

*Conclusion*. Therefore, TA-NRP is morally permissible.

Let's first focus on the fourth premise and consider whether brain death is artificially hastened in TA-NRP. Assume that brain death does not occur following the hands-off period, and physicians ensure its occurrence by clamping arteries to prevent blood flow to the brain. This sounds like artificially hastening brain death, which, according to the ACP, does not happen in standard DCD. However, not all agree with the ACP. Parent et al. 2022, 1308) maintain that cold perfusion with abdominal aortic clamping during standard DCD also artificially hastens brain death, similar to the occlusion of cerebral vessels in TA-NRP:

[A]fter the 5-minute hands-off period, the aorta is clamped at the level of the diaphragm or descending thoracic aorta and the body of the deceased is flushed with ice-cold preservation fluid. The brain’s ischemic time is thus accelerated. In addition to preventing further warm ischemic injury of the transplantable organs, this maneuver ensures complete cessation of brain function prior to removing the organs.

If this is true, it seems that the fourth premise is neither true for standard DCD nor for TA-NRP. Therefore, we might have to conclude that both standard DCD and TA-NRP are morally impermissible. Yet, the ACP and other opponents of TA-NRP do not make such a claim. Why? There seem to be four options: (1) Parent et al.’s claim is incorrect; that is, neither cold perfusion with abdominal aortic clamping in standard DCD nor occluding cerebral vessels in TA-NRP accelerates brain death, because brain death has already occurred; (2) Parent et al.’s claim is correct, and we should conclude that both standard DCD and TA-NRP are morally problematic; (3) Parent et al.’s claim is correct, but opponents have a different (and stronger) argument to show the moral difference between the two procedures; (4) Parent et al.’s claim is correct; however, despite this, both standard DCD and TA-NRP remain morally permissible.

Let’s explore the third option. Perhaps the ACP posits a different condition in the first premise: ‘the procedure is morally permissible if brain death is not artificially hastened during the procedure to justify restoring circulation.’ The fourth premise would then hold true for standard DCD but not for TA-NRP: ‘In standard DCD, brain death is not artificially hastened to justify restoring circulation.’ Such a revision in the opponent’s argument would suggest a difference in intentions between the two procedures and seem to invoke the *Doctrine of Double Effect*.^22^ While the intention in standard DCD is to perfuse organs with cold preservation fluid, the intention in TA-NRP is to directly prevent blood flow to the brain to avoid legal complications when circulation is restarted. It may be true that physicians cannot intend to resuscitate the patient in TA-NRP, but they may well intend to prevent blood flow to the brain and accelerate brain death. In standard DCD, however, the artificial acceleration of brain death is seen as a foreseen but *unintended* consequence of cold perfusion with abdominal aortic clamping. Hence, the moral difference.

For the Doctrine of Double Effect to be applicable, an action must produce both good and bad consequences, allowing us to potentially vindicate an action if the bad consequence is not intended but merely foreseen. The good consequence is the availability of more and better organs for transplantation. The assumed bad consequence is the artificial hastening of brain death, when the patient is *already considered dead* due to ‘permanent’ cessation of circulation. However, is this truly a bad consequence? Assuming that preventing brain circulation indeed artificially accelerates brain death, the ACP must provide a compelling moral reason for us to recognize the badness of this outcome under these circumstances.

One possible reason is that artificially hastening brain death to justify restoring circulation constitutes using the patient as a mere means, thereby violating their autonomy. Admittedly, the patient’s ethically deceased body remains a subject of harm and moral violation. For example, should physicians perform actions on a patient’s deceased body that contravene the preferences expressed by the patient before death or the surrogates’ preferences, it would constitute using them as mere means and breach the principle of respect for autonomy. Or, if someone who is ethically uncomfortable with TA-NRP is forced to undergo the procedure, this would be morally impermissible. However, as long as informed consent is obtained, the procedure itself does not appear to involve using people as mere means or violating their autonomy, unless a compelling reason that we have overlooked forges the link between ‘occluding cerebral circulation of a deceased patient who previously consented to the procedure’ and ‘disrespecting autonomy.’ Even if we accept that physicians manipulate circumstances to meet legal criteria for organ donation, this does not necessarily imply disrespect for autonomy, provided (a) those legal criteria prevent morally permissible and beneficial actions, and (b) the patient and/or surrogates make a conscious and informed decision regarding TA-NRP and the withdrawal of life-sustaining treatments.

Another possible reason is that restarting circulation in TA-NRP is morally objectionable. Once this is established, it could further be argued that artificially hastening brain death to make this morally objectionable action seem less objectionable, by attempting to provide a legal justification, is also wrong. The idea is that the occlusion of cerebral vessels derives its wrongness from the wrongness of restarting circulation because of their connection in TA-NRP. This idea works, for example, when a petrochemical company exploits a legal loophole, resulting in toxic air pollution that causes environmental damage and health problems for nearby communities, and then argues that it adhered to national emissions regulations and permits. Here, both causing air pollution and the attempt at legal justification appear to be wrong. However, it is not as easy to say this about restoring circulation. As previously articulated, we lack solid moral reasons to deem the restoration of circulation objectionable. At best, we can cite legal reasons for claiming it is wrong, which may indeed highlight that physicians are seeking a legal justification for restoring circulation by preventing brain circulation. It is not unreasonable to assert that physicians occlude cerebral vessels primarily to prevent any potential minuscule brain activity due to reperfusion, which could cause legal issues by violating the irreversibility requirement. However, if physicians are seeking legal justification for a morally permissible action, there is no reason to consider this justification morally wrong.

It could be argued that occluding cerebral circulation is indeed an attempt to justify an otherwise morally wrong action because, without such occlusion, the brain would be reperfused, potentially leading to morally significant brain activity. For instance, if reperfusion led to some level of awareness or pain perception, the procedure would contravene the Dead Donor Rule. Although the primary intention of physicians might be to prevent minimal, clinically insignificant brain activity to circumvent potential legal complications arising from the irreversibility requirement, the possibility of morally relevant brain activity due to reperfusion could not be entirely ruled out. This may present a risk, albeit extremely low, of resuscitating the patient before organ procurement. However, could this not constitute a moral reason in favor of preventing brain reperfusion, since it aims to ensure the cessation of neuronal activity and prevent possible awareness or pain perception in a patient whose circulatory death has already occurred? Could this not be viewed as an act of non-maleficence? Technically, such occlusion is not absolutely necessary for restoring circulation; nonetheless, if there is any risk of morally relevant brain activity, the principle of non-maleficence creates a moral obligation for physicians to prevent brain reperfusion.

What about the ACP’s claim that occluding cerebral circulation disrupts the natural course of death? The studies previously mentioned indicate that auto-resuscitation after the five-minute hands-off period is highly improbable. This implies an absence of meaningful cardiac activity capable of sustaining any brain function following the hands-off period in both procedures. By the end of the hands-off period, ‘natural’ circulation to the brain has already ceased, and the brain has been deprived of oxygen for a period most likely resulting in extensive cell death and the loss of all brain functions. Therefore, it seems that, except in exceedingly rare cases, brain death has already occurred prior to the occlusion of cerebral circulation in TA-NRP and the cold perfusion with abdominal aortic clamping in standard DCD. If this is indeed the case, the claim that occluding cerebral circulation goes against the natural course of death or accelerates brain death substantially loses its strength.

It appears that the ACP simply assumes that occluding cerebral circulation in TA-NRP leads to a morally bad consequence, without offering a compelling reason for this belief. Consequently, employing the Doctrine of Double Effect offers little aid to the critics of TA-NRP, allowing us to dismiss the third option—that Parent et al.’s claim is accurate, yet opponents possess a different and stronger argument demonstrating a moral distinction between the two procedures. Additionally, the studies I referenced earlier suggest that brain death has already occurred naturally before the cold perfusion in standard DCD and the occlusion of cerebral reperfusion in TA-NRP. Therefore, the most plausible conclusion is that neither cold perfusion in standard DCD nor occluding cerebral reperfusion in TA-NRP hastens brain death (the first option).

There remains, however, a theoretical possibility that cold perfusion or occlusion of cerebral vessels could accelerate brain death, provided that not all brain functions have been lost when the hands-off period is over. Therefore, I will also consider the second option—that Parent et al.’s claim is correct and both procedures are morally problematic—and the fourth option—that Parent et al.’s claim is correct but both procedures remain morally permissible. I see no strong reason to endorse the second option, while there are compelling reasons favoring the fourth option. First, both the occlusion of cerebral vessels and cold perfusion with abdominal aortic clamping take place after ‘natural’ circulatory death, even assuming they expedite the cessation of all brain functions. Thus, disruption of the ‘natural’ course of death is less of an issue. Second, I have already demonstrated that the potential invalidation of death declaration due to restoration of circulation in TA-NRP is merely a legal issue, not a moral one. Third, we might view these instances of accelerated brain death as acts of non-maleficence since they seem to ensure the cessation of neuronal activity and prevent possible awareness or pain perception. Thus, even if in exceedingly rare instances cold perfusion and the occlusion of brain circulation could indeed accelerate brain death, both standard DCD and TA-NRP remain morally permissible. The ACP’s condition, namely that ‘the procedure is morally permissible if brain death is not artificially hastened during the procedure (to justify restoring circulation),’ should not be included in the first premise to begin with.

Lastly, let’s consider the question I raised at the beginning of this section: How can we claim irreversible cessation of all brain functions if we must actively prevent their potential resumption? This question underscores, once again, the importance of recognizing that ‘permanence’ rather than ‘irreversibility’ better reflects medical practice. As with circulatory death, the idea here is that all brain functions have ‘permanently’ ceased, not that it is absolutely impossible for some brain functions to be restored. In fact, Peled et al. (2022) have contended that occluding cerebral vessels may not entirely exclude all circulation to the brain, suggesting that it may be inappropriate to speak of the ‘impossibility’ of reperfusion in TA-NRP.^23^ Similarly, as in the permanent loss of circulatory functions, the key factor is the absence of any intention or attempt to resuscitate the patient. This stance could also involve actively preventing (theoretically possible) resuscitation throughout the organ donation process. While the occlusion of brain circulation is primarily undertaken to circumvent potential legal ramifications associated with clinically insignificant brain activity, an exceedingly small theoretical risk of restoring morally significant brain functions, such as awareness or pain perception, may persist without occlusion. Yet, there is no intention to resuscitate the patient in TA-NRP. Quite the contrary, occluding cerebral vessels further lowers the already negligible probability of any morally relevant brain activity.

There is no good reason to suggest that TA-NRP is morally impermissible. There seems to be no moral difference between TA-NRP and standard DCD, except that TA-NRP results in saving more lives, improving the quality of life for a greater number of individuals, and providing comfort to a larger number of donor families. Therefore, our conclusion should be stronger: TA-NRP is not only morally permissible, but also morally required where it is financially and technically feasible.

## Insights and other objections: non-maleficence, autonomy, and justice

Detractors fail to prove TA-NRP’s moral impermissibility despite its benefits, but they offer two insights to ensure that TA-NRP fully adheres to all bioethical principles. First, additional brain blood flow and activity monitoring and contingency plan to abort the organ procurement may be needed for TA- NRP to avoid awareness or pain perception, *if* it is proven that occlusion of cerebral vessels may not completely exclude all brain flow (Peled et al. 2022, 1646). That is, if a sign of morally relevant brain activity is detected, the procedure should be halted. Nevertheless, *contra* Peled et al., a similar risk is also present in standard DCD, as it is not impossible for auto-resuscitation to occur after death declaration. This extra step would ensure non-maleficence for both DCD procedures.

Second, some supporters of TA-NRP believe families have the right not to receive “too clinical or burdensome” information or to receive it only upon request (Parent et al. 2022, 1309). This view, however, may overlook the importance of informed consent in organ donation. Although informed consent is not sufficient to justify unethical practices,^24^ it is often *necessary* for the moral permissibility of many medical practices, including organ donation. Firstly, autonomy is deeply linked to informed consent, as individuals can truly exercise autonomy only when fully informed about their choices. Secondly, providing information only upon inquiry reduces transparency. Thirdly, some families may not know what questions to ask, potentially leading to decisions made without full understanding. Extending the ‘right not to know’ to organ donation may therefore risk a broader violation of the principle of respect for autonomy than does providing unrequested information about the procedure. (Of course, to ensure comprehensive understanding, the details of the procedure must be communicated in simple language accessible to patients and/or families, ideally by trained medical professionals.) Thus, patients and/or families should receive detailed yet simplified information about the procedure, premortem interventions, and ethical controversies, which is essential for establishing public trust in organ donation. Moreover, further independent moral evaluations of TA-NRP may be needed to alleviate concerns about possible conflicts of interest (DeCamp et al. 2022b, 293; Opole et al. 2022).

Regarding justice, there is not much to be said because ACP’s argument is misplaced. Social determinants of health (e.g., income, education, housing, etc.) and discrimination create unfair health inequalities (Daniels 2008, ch. 3), and drug overdose victims are often of lower socioeconomic status. However, it is not clear how choosing standard DCD over TA- NRP will address inequities or their sources. It is difficult to pinpoint the precise reason(s) the ACP has in mind that suggest TA-NRP poses additional justice issues beyond existing inequities. They claim that TA-NRP exacerbates the inequitable distribution of benefits and burdens within society without providing further details. It is, however, difficult to see why this would be the case, provided that drug overdose victims and/or their families make voluntary decisions about organ donation. Moreover, organ donation might bring a measure of relief to families of drug overdose victims, helping alleviate the grief of their loss with the prospect that the victim’s organs will save others’ lives. As TA-NRP produces more and better-quality organs, it can offer comfort to an increased number of donor families over standard DCD.

Finally, ex situ perfusion techniques both complicate the process and increase the costs of organ procurement (Adams et al. 2022, 2304), and they seem to be less effective at protecting organs than TA-NRP. First, procuring multiple organs requires multiple machines, which substantially raises costs and the probability of insurance rejections. Second, the shortage of machines and staff for all donations could lead to further justice issues, as availability is likely to be limited to certain areas. Additionally, ex situ alternatives may not be feasible for cardiac death patients due to the rapid onset of tissue death and clotting in the absence of blood flow. This may prevent the procurement of all otherwise donatable organs. Thus, ex situ techniques might not be as effective as TA-NRP in preserving organs, particularly those that are more susceptible to ischemic injury after circulatory death, such as the heart.

## Conclusion

TA-NRP not only seems ethically acceptable but also ethically required where it is financially and technically feasible, as it provides more and better organs for those in need, save more lives, and advances medical research. Detractors must show that it is deeply problematic compared to standard DCD to justify stopping it, but they have not been able to do so. Provided that informed consent has been provided regarding the withdrawal of life-saving treatments and organ donation, medical futility is determined according to accepted medical standards, extra steps are taken to ensure non-maleficence, and patients and/or surrogates are given detailed information about the procedure and ethical debates surrounding it before giving consent, there appears to be no serious ethical issues with TA-NRP, which upholds all bioethical principles.

## Data Availability

No new data, materials, or code were created or used in the production of this manuscript. All discussions and analyses are derived from previously published studies, which are duly cited and referenced in the text.

## Footnotes

1 Organ donation is ‘controlled’ when the withdrawal of life-sustaining treatments is planned following the consent of the patient and/or their family.

2 When procurement of the heart is intended, the ascending aorta is also clamped to introduce cardioplegia—a solution that stops the heart’s contractions and protects it from ischemic injury during the period of ceased blood flow.

3 This is because, according to the ACP, physicians cannot procure organs using the UDDA’s circulatory death criterion once circulation has been restored. Therefore, the ACP believes that physicians intentionally cause brain death and procure organs according to the brain death criterion.

4 Following the same reasoning, we cannot justify the procurement of organs from a healthy individual to save multiple patients by invoking the utilitarian value of such an action, because the principle of respect for persons and their inherent dignity holds lexical priority over the principle of beneficence or utility. That is, no matter how beneficial the outcomes might be in terms of utility, these outcomes cannot be pursued if doing so would violate the principle of respect for individuals.

5 See Oxford University Press. 2024. “resuscitation, n.” OED Online. Last modified July 2023. https://www.oed.com/dictionary/resuscitation_n?tab=meaning_and_use.

6 There have been few cases in history where patients were ‘resurrected’ after the hands-off period of five minutes due to auto-resuscitation, leading to the use of the term ‘Lazarus phenomenon’ for these rare instances (Sahni 2016). Since ‘unachievable’ does not imply practical impossibility but rather suggests high improbability, this statement remains accurate.

7 Armstrong's paper focuses on how speakers use language to convey their mental states, thereby providing an analysis of linguistic meaning. Importantly, the distinction between objectives and intentions he proposes may have broader applications, extending to metaphysical differences in mental states associated with planning, intending, or aiming. This distinction thus not only aids in linguistic analysis but also enriches our understanding of the psychological dimensions of human goal-setting and action-planning.

8 It is worth noting that Armstrong’s distinction between objectives and intentions is not necessary to argue that physicians *do not* intend to resuscitate the patient in TA-NRP. This distinction only reinforces the claim that one *cannot* intend what one knows to be unachievable. Nevertheless, even if it were conceivable for physicians to intend to resuscitate patients in TA-NRP, it would be unreasonable to suggest that they actually intend to keep the patient *alive and functioning* during organ transplantation—a view no detractor would likely endorse. I believe this scenario would represent a morally relevant sense of ‘resuscitation’ that violates the Dead Donor Rule. As we will see, detractors seem to conflate the morally and legally relevant senses of ‘resuscitation.’.

9 It is unclear to me whether opponents believe that clinically insignificant circulatory activity justifies aborting the procedure. As I understand it, when DeCamp and his colleagues refer to ‘auto-resuscitation,’ they exclude clinically insignificant activity that fails to restore sustained heart function and circulation capable of supporting life. Nonetheless, they oppose restoring circulation in TA-NRP, which appears clinically insignificant, as the patient is already brain dead prior to restoration. (Brain death will be discussed later.) Furthermore, Glazier and Capron assert that medical futility is irrelevant in determining death. However, wouldn’t this assertion apply not only to the resumption of circulation in TA-NRP but also to any clinically insignificant circulatory activity following the hands-off period in standard DCD that would make medical interventions futile? In any case, as I argue below, clinically insignificant activity does not seem to provide sufficient moral grounds to terminate either procedure.

10 As proposed by Beauchamp and Childress (2013), the principles of beneficence, non-maleficence, autonomy, and justice are the core principles of bioethics.

11 It could be claimed that minor cardiac electrical activity or agonal contractions, which do not sustain vital body functions or restore some level of consciousness or sentience, should *not* be considered *clinically meaningful* ‘circulatory function’ that violates the irreversibility requirement for death determination. (Thus, the argument would proceed, it is unnecessary for me to attempt to prove that continuing with the organ donation process after a clinically insignificant resumption of circulatory function is morally permissible.) However, if this claim is accepted, then the fact that the circulation in TA-NRP is not clinically meaningful, thereby rendering any medical intervention futile, would indicate that TA-NRP does not violate the UDDA. Moreover, if the UDDA’s irreversibility requirement pertains only to ‘clinically meaningful’ circulatory functions that might prevent medical interventions from being futile, this appears to contradict the opponents’ position that medical futility is irrelevant in determining the validity of a death declaration.

12 This translation of Kant is taken from *The Cambridge Edition of the Works of Immanuel Kant*, edited by Paul Guyer and Allen Wood (1992). Page numbers refer to the volume: page of the Academy edition of *Kants Gesammelte Schriften* (De Gruyter 1900).

13 Although informed consent is an important component of not treating someone solely as a means, it is not a *sufficient* condition. Kant’s Formula of Humanity stresses the imperative of treating humanity (our rational nature) never as a mere means but always as an end in itself (*Groundwork of the Metaphysics of Morals*, 4:429). This principle asserts that we, by virtue of our freedom, are not mere instruments of nature or means to its ends. Instead, we—our rational nature, to be more specific—possess the capability to legislate our own laws and are, therefore, ends in ourselves. Moral principles emerge from our rational nature rather than from actual preferences based on desires and inclinations. Therefore, even if one gives their informed consent to an action that is deemed wrong by our rational nature, such as slavery, cannibalism, or female genital mutilation, the action still remains wrong.

14 Again, I assume that informed consent of the patient or surrogate(s) has been obtained. Although the patient is regarded as ethically deceased, it would be incorrect to assert that the body cannot be subjected to harm or moral violation because it is not a case involving a moral agent. It would be morally objectionable for physicians to do whatever they wish with that body. At that point, the patient’s previous autonomous claims, as expressed through an advance directive or living will, or the consent of the patient’s family, continue to create moral obligations for the physicians.

15 A Kantian reason against harming animals could roughly take the form of, ‘If you harm animals, you will be more inclined to harm humans as well.’.

16 In Switzerland, Norway, New Zealand, and the Italian city of Reggio Emilia only, it is illegal to boil lobsters alive without stunning them first (Millstein 2023).

17 This condition implies that auto-resuscitation will not occur within the specific circumstances being considered. More precisely, it is *anticipated* that auto-resuscitation will not take place following the standard hands-off period of five minutes. This is distinct from the ACP’s condition stating that ‘auto-resuscitation is not *possible*,’ which seems to imply a physical impossibility of auto-resuscitation under any conditions, once the hands-off period is over.

18 Note that the second condition looks identical to one of ACP’s initial conditions. Here, however, the term ‘resuscitation’ is used in its morally relevant sense, consistent with its interpretation in medical practice, and does not encompass any additional legal connotations that do not align with or are not applied in medical practice.

19 Parent et al. do not mention the possibility of an unassisted return of clinically insignificant circulatory function when they describe the ‘permanence’ condition. However, I believe that including this information complements the condition, considering the ACP’s emphasis on ‘impossibility’ in relation to the ‘irreversibility’ requirement.

20 This review only includes articles that report cases where spontaneous circulation resumed after cardiopulmonary resuscitation (CPR) was stopped.

21 The same point would, of course, hold for the resumption of clinically insignificant brain activity or function as well.

22 According to the Doctrine of Double Effect, if (a) an action has both good and bad consequences, (b) the bad consequences are not intended but merely foreseen, and (c) the good consequence outweighs the bad one, then the act is permissible. See Vaughn (2017, 40–42).

23 However, as I have argued, a clinically insignificant resumption of activity or function after death declaration does not render the procedure morally impermissible.

24 For example, experimenting with unproven and potentially dangerous drugs without adequate safety testing would be unethical, even if participants gave their informed consent to participate.

## Declarations

### Animal welfare

Not applicable, as the research did not involve animals.
